# Factor-Based Extrusion Timing of Ventilation Tubes in Otitis Media with Effusion: A Survival Analysis

**DOI:** 10.3390/medicina62071376

**Published:** 2026-07-17

**Authors:** Kadir Sinasi Bulut, Fatih Gul, Serkan Şerifler, Burak Celik, Muhammed Furkan Aydogdu, Selman Seckin

**Affiliations:** 1 Head and Neck Surgery, Department of Otolaryngology, Faculty of Medicine, Ankara Yildirim Beyazit University, 06760 Ankara, Turkey; kadirsinasibulut@gmail.com (K.S.B.); serkanserifler@gmail.com (S.Ş.); burakcelik70@gmail.com (B.C.); 2 Head and Neck Surgery, Department of Otolaryngology, Faculty of Medicine, Lokman Hekim University, 06510 Ankara, Turkey; 3 Head and Neck Surgery, Department of Otolaryngology, Ankara Bilkent City Hospital, 06800 Ankara, Turkey; m.furkan.aydogdu@gmail.com (M.F.A.); slmnsckn34@gmail.com (S.S.)

**Keywords:** ventilation tube, otitis media with effusion, tube extrusion time, survival analysis, Cox proportional hazards regression, Kaplan–Meier, middle ear effusion, tympanostomy tube, tube obstruction, pediatric otolaryngology

## Abstract

*Background and Objectives*: Ventilation tube insertion (VTI) is the most commonly performed surgical procedure for otitis media with effusion (OME) in children. While the factors associated with tube extrusion have been investigated, their quantitative impact on the timing of extrusion remains insufficiently characterized. This study aimed to identify independent factors associated with ventilation tube extrusion time and to quantify the magnitude and direction of their effect using survival analysis methodology. *Materials and Methods*: This retrospective cohort study included 150 patients (262 ears) who underwent VTI with Shepard-type tubes for OME at a tertiary referral center between February 2019 and July 2025. Patient-level variables (age, sex, laterality, allergic rhinitis, secondhand smoke exposure, adenoidectomy, and tonsillectomy) were analyzed per patient (*n* = 150), while ear-specific variables (effusion type, history of previous VTI, and tube obstruction) were analyzed per ear (*n* = 262). Univariate and multivariate Cox proportional hazards regression analyses were performed to identify independent factors associated with extrusion time. Kaplan–Meier survival curves with log-rank tests were used to compare median extrusion times between groups. *Results:* The mean ventilation tube extrusion time was 280.38 ± 99.24 days (9.35 ± 3.30 months). Three independent factors significantly associated with shorter extrusion time were identified in multivariate analysis: tube obstruction during follow-up (HR = 2.629, 95% CI: 1.731–3.993, *p* < 0.001), history of previous VTI (HR = 2.292, 95% CI: 1.678–3.131, *p* < 0.001), and serous effusion type compared to glue (HR = 1.914, 95% CI: 1.455–2.517, *p* < 0.001). Age, sex, laterality, allergic rhinitis, secondhand smoke exposure, adenoidectomy, and tonsillectomy were not significantly associated with extrusion time. *Conclusions:* History of previous VTI, serous effusion type, and tube obstruction during follow-up were independently associated with shorter ventilation tube extrusion time. Notably, tube obstruction during follow-up, which has not been previously evaluated as a factor associated with extrusion time in survival analyses of tube retention, demonstrated the strongest independent effect on extrusion timing. These findings may assist clinicians in anticipating tube behavior and individualizing postoperative follow-up schedules.

## 1. Introduction

Otitis media with effusion (OME) is one of the most common pediatric diseases worldwide, affecting approximately 90% of children by the age of five years, with peak incidence occurring between 2 and 6 years of age [1]. The condition is characterized by the accumulation of fluid in the middle ear cavity in the absence of signs or symptoms of acute infection and represents a leading cause of conductive hearing loss during early childhood. Persistent OME can adversely affect speech and language development, academic performance, and overall quality of life, particularly when left untreated during critical developmental periods [2].

Ventilation tube insertion (VTI), also known as tympanostomy tube placement, is among the most frequently performed surgical procedures in pediatric otolaryngology and remains the standard of care for chronic or recurrent OME that fails to resolve spontaneously [3]. By restoring middle ear aeration and facilitating drainage of effusion, VTI effectively improves hearing thresholds and reduces the burden of recurrent disease [2,3]. In the United States alone, approximately 667,000 tympanostomy tube procedures are performed annually on children younger than 15 years of age, underscoring its widespread clinical relevance [4].

The duration that a ventilation tube remains functional within the tympanic membrane is a critical determinant of treatment success. Premature extrusion may lead to recurrence of middle ear effusion and necessitate reoperation, whereas prolonged retention is associated with an increased risk of complications including persistent tympanic membrane perforation, chronic otorrhea, and tympanosclerosis [5,6]. These clinical challenges have prompted considerable research interest in developing novel tube designs—including dissolvable on-command tympanostomy tubes—aimed at optimizing retention duration while minimizing complications [7]. Identifying the factors that influence tube extrusion time therefore carries important clinical implications: it may enable individualized postoperative follow-up scheduling with closer intervals for patients at higher risk of early extrusion, facilitate more accurate preoperative counseling of families regarding the expected duration of tube function and the potential need for reoperation, inform tube type selection in future procedures for patients with a history of premature extrusion, and encourage active surveillance for tube patency during follow-up visits.

Several studies have investigated the factors associated with ventilation tube extrusion, reporting variable and often inconsistent findings [8,9]. Tube design and material, history of previous tube insertion, characteristics of middle ear effusion, patient age, and concurrent surgical procedures such as adenoidectomy have been proposed as potential determinants; however, no consensus has been reached [8,9]. Although several prior studies have evaluated factors associated with tube extrusion using survival-analysis methods, including Kaplan–Meier estimation and Cox regression [5,8,9], the specific contribution of certain clinically relevant covariates—particularly postoperative tube obstruction—has not been systematically examined. This distinction is clinically meaningful, as the duration of tube retention, rather than simply whether extrusion occurs, determines the adequacy of middle ear ventilation and the likelihood of disease recurrence [6].

Therefore, the present study aimed to evaluate the association between clinical variables and ventilation tube extrusion time in a homogeneous cohort receiving a single tube model (Shepard-type fluoroplastic tubes), with particular emphasis on postoperative tube obstruction as a covariate not previously included in regression models of extrusion timing. By applying Cox proportional hazards regression and Kaplan–Meier analysis to 150 patients (262 ears) who underwent VTI for OME, this study seeks to identify independent factors associated with extrusion time and to quantify the magnitude and direction of their effect.

## 2. Materials and Methods

### 2.1. Study Design and Setting

This retrospective cohort study was conducted at a tertiary referral center affiliated with a university hospital. The study was approved by the institutional ethics committee, and all procedures were performed in accordance with the Declaration of Helsinki. Medical records of patients who underwent VTI for OME between February 2019 and July 2025 were reviewed.

### 2.2. Participants

*Inclusion criteria*: Patients of any age who underwent VTI for OME with documented preoperative tympanometry findings (Type B or Type C) and persistent middle ear effusion lasting more than three months despite conservative management, accompanied by documented hearing loss on audiometric evaluation, were eligible for inclusion.

*Exclusion criteria*: Patients were excluded from the study if: (1) the type of middle ear effusion (serous or glue) was not recorded in the operative notes; (2) preoperative tympanometry data were absent; (3) follow-up records sufficient to determine tube extrusion time were unavailable; (4) a concomitant craniofacial anomaly was present (e.g., cleft palate, Down syndrome); (5) tympanic membrane retraction or adhesive otitis media was present; (6) the ventilation tube was inserted in a quadrant other than the antero-inferior quadrant of the tympanic membrane; (7) the follow-up duration was insufficient to determine tube extrusion; or (8) operative records contained incomplete information regarding surgical technique, tube placement, or effusion type. After applying inclusion and exclusion criteria, a total of 150 patients were included in the final analysis.

### 2.3. Diagnosis of Otitis Media with Effusion

The diagnosis of OME was established based on otoscopic examination and tympanometry. Tympanometry was classified according to Jerger’s classification; Type B (flat curve) and Type C (negative peak pressure) patterns were considered consistent with middle ear effusion. All patients underwent otoscopic evaluation by an otorhinolaryngologist prior to surgery.

### 2.4. Surgical Procedure

All VTI procedures were performed under general anesthesia in the operating room. Myringotomy was performed in the antero-inferior quadrant of the tympanic membrane using a standard myringotomy knife, followed by aspiration of middle ear effusion and insertion of a Shepard-type ventilation tube. The same tube model was used in all 262 ears throughout the study period: the OSSEOUS Shepard with wire (EON Meditech Pvt. Ltd., Gujarat, India; reference E2114W), a double-flanged grommet-type tube made of fluoroplastic (PTFE) with an inner lumen diameter of 1.14 mm, supplied sterile (ethylene oxide sterilization, CE 1282). The nature of the middle ear effusion encountered at the time of myringotomy was classified intraoperatively by the operating surgeon as either serous (thin, watery fluid) or glue (thick, viscous fluid, including mucoid effusion). In patients who underwent bilateral VTI, the mean extrusion time of both ears was used as the unit of analysis, as all patient-level variables were recorded per patient rather than per ear. Concurrent procedures including adenoidectomy and tonsillectomy were performed at the surgeon’s discretion based on clinical indications and were recorded for analysis.

### 2.5. Follow-Up and Outcome Assessment

Patients were evaluated at regular follow-up visits every two to three months postoperatively. At each visit, otoscopic examination was performed to assess tube position, patency, and tympanic membrane integrity. Tube extrusion time was defined as the number of days elapsed from the date of surgery to the date of tube extrusion. The date of extrusion was calculated as the midpoint between the last clinic visit at which the tube was confirmed to be in situ and the first visit at which the tube was no longer observed in the tympanic membrane, consistent with the methodology described by Song et al. [9]. For ear-level analyses, individual extrusion times were used for each ear. For patient-level analyses, the mean extrusion time of both ears was used in patients who underwent bilateral VTI, as all patient-level covariates were recorded per patient rather than per ear.

The following variables were recorded for each patient: age at the time of surgery, sex, history of previous VTI, type of middle ear effusion (serous vs. glue), laterality (unilateral vs. bilateral), presence of allergic rhinitis, secondhand smoke exposure, concurrent adenoidectomy, concurrent tonsillectomy, postoperative tube obstruction observed during follow-up visits, and development of persistent tympanic membrane perforation following tube extrusion.

Variables were classified as patient-level or ear-level based on the biological level at which each factor operates. Variables that could differ between the two ears of the same patient—effusion type, history of previous VTI, and tube obstruction—were analyzed at the ear level (*n* = 262 ears), whereas systemic variables invariant across ears within a single patient (age, sex, laterality, allergic rhinitis, secondhand smoke exposure, adenoidectomy, and tonsillectomy) were analyzed at the patient level (*n* = 150 patients).

Tube obstruction was defined as complete occlusion of the ventilation tube lumen identified on otoscopic examination during routine postoperative follow-up visits. This binary definition (patent vs. non-patent) is consistent with the approach used in the existing literature [5,10]. When complete tube obstruction was identified, management consisted of microscopic aspiration under otomicroscopy; in cases where mechanical aspiration alone was insufficient, hydrogen peroxide and/or oxygen-boric acid solution was applied to the tube lumen to soften the obstructing material, followed by repeat aspiration. Ears in which tube patency could not be restored despite these interventions were classified as having persistent tube obstruction.

### 2.6. Statistical Analysis

Statistical analyses were performed using IBM SPSS Statistics version 31.0 (IBM Corp., Armonk, NY, USA). Continuous variables are presented as mean ± standard deviation (SD) or median with interquartile range (IQR), as appropriate. Categorical variables are presented as frequencies and percentages. The normality of continuous variables was assessed using the Shapiro–Wilk test.

Survival analysis was performed using the Kaplan–Meier method to estimate cumulative tube retention rates over time. Differences in extrusion time between groups were compared using the log-rank (Mantel–Cox) test. Variables with a *p*-value of less than 0.20 in univariate Cox proportional hazards regression analysis were included in the multivariate model [11]. Results are expressed as hazard ratios (HR) with 95% confidence intervals (CI). The proportional hazards assumption was verified for all variables in the multivariate model using the Schoenfeld residuals test. Statistical significance was set at *p* < 0.05 for all analyses. A generative AI tool (Claude, Opus 4.6, Anthropic, San Francisco, CA, USA) was used solely for language refinement during manuscript preparation. All scientific content, data analysis, interpretations, and conclusions were performed and verified by the authors, who take full responsibility for the accuracy and integrity of the work.

## 3. Results

### 3.1. Patient and Ear Characteristics

A total of 150 patients (262 ears) who underwent ventilation tube insertion for otitis media with effusion were included in the study. The mean age was 5.82 ± 2.55 years, and 86 patients (57.3%) were male. Bilateral OME was present in 112 patients (74.7%). The mean ventilation tube extrusion time was 280.38 ± 99.24 days (9.35 ± 3.30 months), with a median of 282 days (IQR: 213–352 days). Glue effusion was the predominant intraoperative finding (170 ears, 64.9%). Postoperative otorrhea was recorded in 2 patients (1.3%) and was managed conservatively with ototopical antibiotic drops. The complete demographic and clinical characteristics are presented in Table 1. Complete tube obstruction during follow-up was documented in 42 ears (16.0%). Following microscopic aspiration and topical treatment with hydrogen peroxide and/or oxygen-boric acid solution, tube patency was successfully restored in 12 ears (28.6%), while 30 ears (71.4%) remained persistently obstructed. Persistent tube obstruction was used as the binary covariate in subsequent survival analyses.

### 3.2. Univariate Cox Proportional Hazards Regression Analysis

Univariate Cox regression analysis was performed for all candidate variables (Table 2). Three variables demonstrated statistically significant associations with extrusion time: history of previous VTI (HR = 2.251, *p* < 0.001), effusion type (HR = 1.489, *p* = 0.003), and tube obstruction during follow-up (HR = 2.791, *p* < 0.001). Age, sex, laterality, allergic rhinitis, secondhand smoke exposure, adenoidectomy, and tonsillectomy were not significantly associated with extrusion time.

### 3.3. Multivariate Cox Proportional Hazards Regression Analysis

All three variables that reached statistical significance in univariate analysis were entered into the multivariate Cox regression model at the ear level (*n* = 262 ears). All three remained independently associated with extrusion time: history of previous VTI (HR = 2.292, *p* < 0.001), effusion type (HR = 1.914, *p* < 0.001), and tube obstruction during follow-up (HR = 2.629, *p* < 0.001) (Table 3, Model A). The overall model was statistically significant (log-likelihood ratio *p* < 0.001), and the proportional hazards assumption was confirmed for all variables using the Schoenfeld residuals test (all *p* > 0.05). To evaluate whether tube obstruction lies on the causal pathway between effusion type and extrusion time, a cross-tabulation of effusion type and persistent tube obstruction was performed. There was no significant association between the two variables (Pearson χ^2^ = 1.061, *p* = 0.303; Fisher’s exact test, *p* = 0.416). Persistent tube obstruction was observed in 22 of 170 glue ears (12.9%) and 8 of 92 serous ears (8.7%). A sensitivity analysis comparing the full multivariate Cox model (including tube obstruction) with a reduced model (excluding tube obstruction) demonstrated minimal change in the hazard ratio for effusion type (HR = 1.914 vs. HR = 1.748), confirming that the two covariates capture independent effects and that overadjustment is not a concern (Table 3, Model B).

### 3.4. Kaplan–Meier Survival Analysis

**Figure 1:**
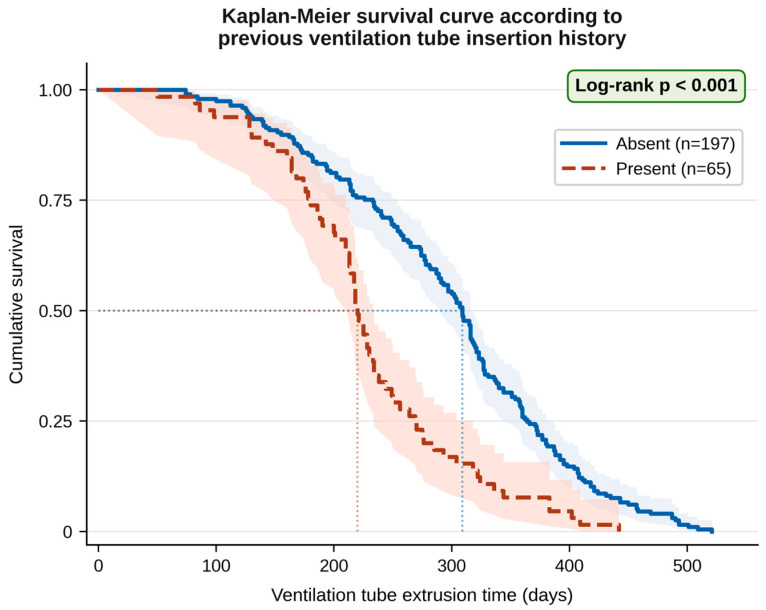
Kaplan–Meier survival curves for ventilation tube extrusion time according to previous VT insertion history. Shaded areas represent 95% confidence intervals. Dotted lines indicate median extrusion time.

**Figure 2:**
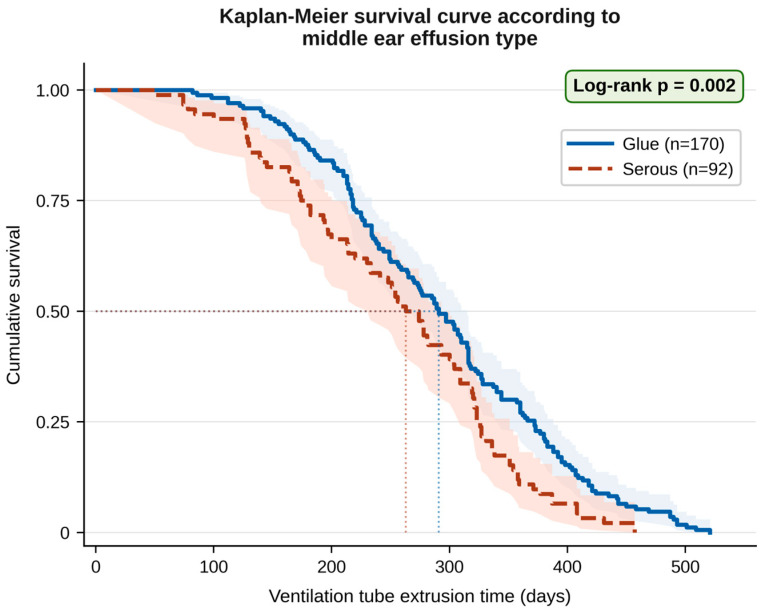
Kaplan–Meier survival curves for ventilation tube extrusion time according to middle ear effusion type. Shaded areas represent 95% confidence intervals. Dotted lines indicate median extrusion time.

**Figure 3:**
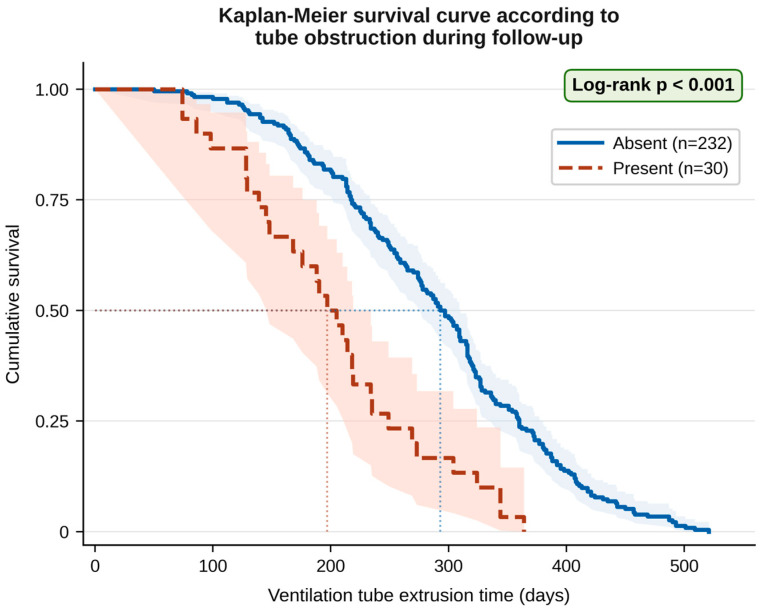
Kaplan–Meier survival curves for ventilation tube extrusion time according to tube obstruction during follow-up. Shaded areas represent 95% confidence intervals. Dotted lines indicate median extrusion time.

Kaplan–Meier survival analysis and log-rank test results are presented in Table 4. Patients without a history of previous VTI had a significantly longer median extrusion time than those with prior insertion (309 vs. 220 days; *p* < 0.001) (Figure 1). Glue effusion was associated with a significantly longer median extrusion time compared to serous effusion (291 vs. 263 days; *p* = 0.002) (Figure 2). Ears without tube obstruction had a significantly longer median extrusion time than those with obstruction (293 vs. 197 days; *p* < 0.001) (Figure 3). No significant differences were observed for sex, laterality, adenoidectomy, tonsillectomy, or allergic rhinitis (Table 4).

## 4. Discussion

The present study investigated the factors influencing ventilation tube extrusion time in 150 patients (262 ears) who underwent VTI for OME, with particular emphasis on quantifying the effect of each variable on the timing of extrusion using Cox proportional hazards regression and Kaplan–Meier survival analysis. Three variables were identified as independently associated with extrusion time: history of previous VTI, intraoperative effusion type, and tube obstruction observed during follow-up. While prior studies have employed survival-analysis approaches to evaluate tube extrusion—including Yoo et al. [5] in a multicenter registry and Otsuka et al. [12] using Kaplan–Meier analysis for T-tubes—the present study adds to this body of evidence by examining a homogeneous cohort treated with a single Shepard-type tube model and by including persistent tube obstruction as a covariate not previously incorporated into regression models of extrusion timing.

The mean ventilation tube extrusion time in our cohort was 280.38 ± 99.24 days (approximately 9.3 months), with a median of 282 days. This finding is broadly consistent with the published literature for Shepard-type tubes, which generally report extrusion times ranging from 6 to 12 months, and is comparable to the mean extrusion time of 254 days reported by Song et al. [9] and the overall mean of 225.85 days reported by Lin et al. [8]. However, the extrusion times observed in our cohort were somewhat longer than those reported in certain prior studies, which may be attributable to differences in patient selection, follow-up protocols, and effusion characteristics across study populations.

### 4.1. History of Previous VTI

The most significant independent factor associated with tube extrusion time in our study was a history of previous VTI (HR = 2.292, *p* < 0.001). Patients with prior tube insertion had a median extrusion time of 220 days, compared to 309 days in those undergoing their first procedure. This finding is consistent with results reported by Song et al. [9], who observed a significantly shorter extrusion time in patients with a prior VTI history (203 days vs. 279 days, *p* = 0.013), and by Lin et al. [8], who similarly identified previous VTI history as a significant predictor of extrusion time in both children and adults. The proposed mechanism underlying this relationship relates to structural changes in the tympanic membrane induced by prior tube insertion. Repeated myringotomy and tube placement may result in scarring, loss of the middle fibrous layer, and alterations in tympanic membrane elasticity, thereby reducing the mechanical resistance to outward epithelial migration and facilitating earlier extrusion [8]. Clinicians should therefore anticipate a shorter tube retention period in patients with a history of prior VTI and consider this factor when planning postoperative follow-up schedules or discussing the likelihood of requiring repeat surgery.

### 4.2. Effusion Type

Intraoperative effusion type was identified as the second independent factor associated with extrusion time in the multivariate model (HR = 1.914, *p* < 0.001). Patients with serous effusion had a significantly shorter median extrusion time compared to those with glue effusion (263 days vs. 291 days). This finding is in agreement with the results of Song et al. [9], who reported the shortest extrusion time in the serous effusion group (190 days) compared to glue (273 days) and mucoid (283 days) groups. The biological basis for this observation likely relates to the viscosity and inflammatory profile of middle ear effusion. Glue effusions, characterized by higher mucin content and increased pro-inflammatory cytokines, are associated with more advanced stages of OME and greater mucosal involvement [9,11,13]. This inflammatory milieu may contribute to a more resistant tympanic membrane environment, thereby delaying tube extrusion. In contrast, serous effusion likely represents an earlier and less inflammatory stage of disease, with relatively preserved Eustachian tube function, which may facilitate faster tympanic membrane healing and earlier extrusion [9]. Notably, Yoo et al. [5] did not observe a significant relationship between effusion composition and tube extrusion time in their multicenter study, though they did identify effusion type as a significant predictor of time to effusion recurrence. Importantly, cross-tabulation revealed no significant association between effusion type and persistent tube obstruction (*p* = 0.303), and the non-significant trend was in the opposite direction, with obstruction being numerically more frequent in glue ears (12.9%) than in serous ears (8.7%) [14]. A sensitivity analysis excluding tube obstruction from the Cox model showed only an 8.7% change in the hazard ratio for effusion type (from 1.914 to 1.748), confirming that effusion type and tube obstruction represent independent pathways influencing extrusion timing rather than a single causal chain. These discordant findings highlight the ongoing lack of consensus in the literature and underscore the need for further investigation using standardized effusion classification criteria.

### 4.3. Tube Obstruction During Follow-Up

Tube obstruction observed during postoperative follow-up was the third independent factor associated with extrusion time in our cohort, and notably demonstrated the highest hazard ratio in the multivariate model (HR = 2.629, *p* < 0.001). Ears in which tube obstruction was detected had a markedly shorter median extrusion time (197 days vs. 293 days). Tube obstruction is a well-recognized complication of VTI, with reported rates ranging from 1.4% to 36.0% and pooled estimates of approximately 7% in meta-analyses [15], consistent with the 5.2–6.0% rates reported by Alvi et al. [14]. The rate of complete tube obstruction in our cohort (42 of 262 ears, 16.0%) is within this reported range. The causes of tube obstruction described in the literature include dried blood clot, inspissated mucoid secretions, cerumen, and epithelial casts, with early obstruction most commonly attributed to intraoperative bleeding and delayed obstruction to inspissated secretions [10,16]. In our cohort, tube patency was restored in 12 of 42 obstructed ears (28.6%) following microscopic aspiration and topical treatment with hydrogen peroxide and/or oxygen-boric acid solution; however, 30 ears (71.4%) remained persistently obstructed despite intervention. Persistent tube obstruction—representing ears in which patency could not be restored—was used as the binary covariate in the survival analysis, and its risk factors, including serous effusion and delayed postoperative follow-up, have been previously characterized [10]. However, while these prior studies investigated factors that predispose to tube obstruction itself, the role of tube obstruction as an independent factor influencing extrusion timing has not been evaluated in survival analyses examining the determinants of tube retention. The studies by Song et al. [9], Lin et al. [8], Alaraifi et al. [17], and Yoo et al. [5]—which represent the primary literature on factors influencing tube extrusion time—did not include tube obstruction as a covariate in their regression models. Conrad et al. [10] demonstrated that tube obstruction is most frequently identified during the early postoperative period, particularly within the first two weeks, and that delayed initial follow-up increases the risk of undetected obstruction. The mechanism by which tube obstruction may accelerate extrusion likely involves perturbation of middle ear ventilation, leading to accumulation of secretions around the tube and inflammatory changes in the tympanic membrane that facilitate outward migration. Alternatively, obstruction may represent a marker of more active middle ear disease, in which ongoing mucosal secretion and inflammation create conditions unfavorable to prolonged tube retention. This finding has potential clinical implications: patients in whom tube obstruction is identified at follow-up visits should be monitored more closely, as they may be at higher risk for premature extrusion and subsequent recurrence of middle ear effusion.

### 4.4. Variables That Did Not Associate with Extrusion Time

Several variables evaluated in this study did not demonstrate a significant association with tube extrusion time, including age, sex, laterality, adenoidectomy, tonsillectomy, allergic rhinitis, and secondhand smoke exposure. The absence of a significant effect of age on extrusion time is consistent with the findings of Song et al. [9], Alaraifi et al. [17], and the multicenter EVENT study by Yoo et al. [5]. Although younger children have been proposed to have different tympanic membrane properties and Eustachian tube function, these differences do not appear to translate into a meaningful difference in extrusion timing for short-term tubes. Adenoidectomy did not significantly influence extrusion time in our study, a finding consistent with results reported by Song et al. [9], Alaraifi et al. [17], and Szekely et al. [18], who demonstrated that although adenoid hypertrophy contributes to OME pathogenesis through mechanical obstruction and mucosal inflammation, concurrent adenoidectomy does not appear to modify the biological process of tube extrusion once middle ear ventilation has been restored. The absence of a significant effect of allergic rhinitis on extrusion time is also consistent with the existing literature [5,17]. While allergic rhinitis contributes to Eustachian tube dysfunction and thereby to the development of OME, its local inflammatory effect on the tympanic membrane appears to be largely neutralized once middle ear ventilation is restored through tube placement.

### 4.5. Limitations

This study has several limitations that should be acknowledged. First, its retrospective design introduces the potential for selection bias and incomplete data ascertainment, despite the application of strict inclusion and exclusion criteria. Second, all patients received Shepard-type ventilation tubes, which precludes comparison across different tube designs and limits the generalizability of findings to other tube types. Third, the classification of effusion type as serous or glue was based on intraoperative visual inspection by the operating surgeon without objective viscosity measurement, introducing a degree of subjectivity. Fourth, the relatively small sample size, particularly for subgroups such as tonsillectomy (*n* = 16), secondhand smoke exposure (*n* = 15), and postoperative otorrhea (*n* = 2, 1.3%), may have limited statistical power to detect associations for these variables; the low rate of postoperative otorrhea in particular precluded meaningful statistical analysis of its association with tube extrusion time. Fifth, a hybrid analytical framework was adopted in which ear-specific variables (effusion type, history of previous VTI, and tube obstruction) were analyzed at the ear level (*n* = 262 ears), whereas patient-level variables (age, sex, allergic rhinitis, adenoidectomy, tonsillectomy, and secondhand smoke exposure) were analyzed at the patient level (*n* = 150 patients); this dual-level approach, while methodologically justified, precludes a single unified multivariate model incorporating both ear- and patient-level covariates. Sixth, as all patients were treated at a single tertiary referral center, the results may not be fully generalizable to other clinical settings. Seventh, the retrospective design precluded systematic subclassification of tube obstruction etiology (e.g., dried blood clot, inspissated secretions, cerumen, or epithelial debris) for each individual case, limiting our ability to assess whether specific obstruction subtypes differentially affect extrusion timing. Eighth, the follow-up interval of two to three months introduces an inherent imprecision of approximately ±15–22 days in the estimation of the exact extrusion date using the midpoint method; however, this non-differential measurement error applies equally across all comparison groups and is unlikely to have introduced systematic bias, particularly given that the observed between-group differences in median extrusion time (ranging from 28 to 96 days) substantially exceeded this margin of imprecision. Finally, as all patients were considered to have experienced tube extrusion, censored observations were not incorporated into the survival analysis, which represents a methodological limitation relative to studies with formal censoring based on documented follow-up duration.

## 5. Conclusions

In conclusion, this retrospective study demonstrates that history of previous VTI, serous effusion type, and tube obstruction during follow-up were independently associated with shorter ventilation tube extrusion time in patients with OME. Among these, tube obstruction during follow-up demonstrated the strongest effect (HR = 2.629), followed by history of previous VTI (HR = 2.292) and serous effusion type (HR = 1.914). History of previous VTI, associated with a more than two-fold increase in extrusion hazard, suggests that structural alterations of the tympanic membrane from prior surgery may accelerate the natural extrusion process. Serous effusion, reflecting an earlier and less inflammatory stage of middle ear disease, was similarly associated with significantly faster extrusion compared to glue effusion, underscoring the role of effusion viscosity and mucosal involvement in determining tube retention. Tube obstruction during follow-up, which has not been previously examined as a determinant of extrusion timing in survival analyses of tube retention, highlights the clinical importance of active surveillance for tube patency during postoperative visits. By examining these associations in a homogeneous cohort treated with a single tube model and incorporating tube obstruction as a previously unexamined covariate, these findings may assist clinicians in anticipating tube behavior and individualizing postoperative follow-up schedules.

## Figures and Tables

**Table 1:** Demographic and clinical characteristics of patients who underwent ventilation tube insertion for otitis media with effusion.

Variable	Category/Statistic	*N* (%) or Mean ± SD
Age, year	Mean ± SD	5.82 ± 2.55
	Median (IQR)	5 (4–7)
	Min–Max	1–12
Sex	Male	86 (57.3%)
	Female	64 (42.7%)
Ventilation Tube Extrusion Time (VTT)	Mean ± SD (days)	280.38 ± 99.24
	Median (IQR) (days)	282 (213–352)
	Mean ± SD (months)	9.35 ± 3.30
	Min–Max (days)	50–521
Laterality	Bilateral	112 (74.7%)
	Unilateral	38 (25.3%)
Type of effusion	Glue	170 (64.9%)
	Serous	92 (35.1%)
History of ventilation tube insertion	Yes	65 (24.8%)
	No	197 (75.2%)
Adenoidectomy	Performed	92 (61.3%)
	Not performed	58 (38.7%)
Tonsillectomy	Performed	16 (10.7%)
	Not performed	134 (89.3%)
Allergic rhinitis	Present	46 (30.7%)
	Absent	104 (69.3%)
Asthma	Present	3 (2.0%)
	Absent	147 (98.0%)
Secondhand smoke exposure	Yes	15 (10.0%)
	No	135 (90.0%)
Tube obstruction during follow-up	Yes	30 (11.5%)
	No	232 (88.5%)
Post-tube perforation	Yes	8 (3.1%)
	No	254 (96.9%)

Demographic and surgical variables are reported per patient (*n* = 150). Effusion type, history of ventilation tube insertion, tube obstruction, post-tube perforation, and ventilation tube extrusion time are reported per ear (*n* = 262 ears). Tube obstruction refers to persistent obstruction (*n* = 30) in which tube patency could not be restored after intervention; see Section 3.1 for details.

**Table 2:** Univariate Cox proportional hazards regression analysis of factors associated with ventilation tube extrusion time.

Variable	*n*	HR	95% CI	*p*-Value
Age, year	150	1.042	0.975–1.113	0.23
Sex	150	1.013	0.731–1.404	0.94
History of ventilation tube insertion	262	2.251	1.683–3.011	<0.001
Type of effusion (Glue vs. serous)	262	1.489	1.149–1.930	0.003
Laterality (bilateral vs. unilateral)	150	0.956	0.659–1.385	0.811
Allergic rhinitis	150	0.926	0.652–1.315	0.667
Secondhand smoke exposure	150	1.185	0.692–2.029	0.536
Adenoidectomy	150	0.879	0.629–1.228	0.45
Tonsillectomy	150	0.826	0.490–1.392	0.473
Tube obstruction during follow-up	262	2.791	1.886–4.130	<0.001

HR: Hazard Ratio; CI: Confidence Interval. HR > 1 indicates faster extrusion; HR < 1 indicates delayed extrusion.

**Table 3:** Multivariate Cox proportional hazards regression analysis of independent factors associated with ventilation tube extrusion time: full model and sensitivity analysis (*n* = 262).

Variable	Model A (Full Model)			Model B (Sensitivity Analysis)		
	HR	95% CI	*p* Value	HR	95% CI	*p* Value
History of ventilation tube insertion	2.292	1.678–3.131	<0.001	2.581	1.912–3.485	<0.001
Effusion type (Glue vs. serous)	1.914	1.455–2.517	<0.001	1.748	1.338–2.284	<0.001
Tube obstruction during follow-up	2.629	1.731–3.993	<0.001	-	-	-

HR: Hazard Ratio; CI: Confidence Interval; Variables with *p* < 0.20 in univariate analysis were included in the multivariate model. Analysis was performed at the ear level (*n* = 262 ears). The proportional hazards assumption was verified using the Schoenfeld residuals test (all *p* > 0.05). Model A includes all three significant covariates. Model B excludes tube obstruction to evaluate potential overadjustment due to a hypothesized causal pathway between effusion type and tube obstruction; the minimal change in HR for effusion type (1.914 vs. 1.748) confirms that the two covariates capture independent effects.

**Table 4:** Kaplan–Meier survival analysis and log-rank test results for ventilation tube extrusion time according to clinical and demographic variables.

Variable	Group		Median Extrusion Time (Days)	95% CI (Days)	Log-Rank *p*
History of ventilation tube insertion	Absent	197	309	296–322	<0.001
	Present	65	220	211–229	
Effusion type	Glue	170	291	268–314	0.002
	Serous	92	263	238–288	
Tube obstruction during follow-up	Absent	232	293	275–311	<0.001
	Present	30	197	167–227	
Laterality	Unilateral	38	273	205–341	0.811
	Bilateral	112	287	255–319	
Adenoidectomy	No	58	287	232–342	0.448
	Yes	92	280	253–307	
Tonsillectomy	No	134	287	255–319	0.471
	Yes	16	273	216–330	
Allergic rhinitis	Absent	104	287	257–316	0.665
	Present	46	278	214–342	
Sex	Male	86	274	248–300	0.939
	Female	64	302	243–361	

CI: Confidence Interval; VT: Ventilation Tube. Median extrusion time was estimated using the Kaplan–Meier method. Groups were compared using the log-rank (Mantel–Cox) test.

## Data Availability

The data presented in this study are available on request from the corresponding author. The data are not publicly available due to privacy and ethical restrictions.

## Abbreviations

The following abbreviations are used in this manuscript:

OME	Otitis media with effusion
VTI	Ventilation tube insertion
VT	Ventilation tube
HR	Hazard ratio
CI	Confidence interval
IQR	Interquartile range
SD	Standard deviation
KM	Kaplan–Meier

## References

[1] Rosenfeld R.M., Shin J.J., Schwartz S.R., Coggins R., Gagnon L., Hackell J.M., Hoelting D., Hunter L.L., Kummer A.W., Payne S.C. (2016). Clinical Practice Guideline: Otitis Media with Effusion (Update). Otolaryngol. Head. Neck Surg..

[2] Sanyaolu L.N., Cannings-John R., Butler C.C., Francis N.A. (2020). The effect of ventilation tube insertion on quality of life in children with persistent otitis media with effusion. Clin. Otolaryngol..

[3] MacKeith S., Mulvaney C.A., Galbraith K., Webster K.E., Connolly R., Paing A., Marom T., Daniel M., Venekamp R.P., Rovers M.M. (2023). Ventilation tubes (grommets) for otitis media with effusion (OME) in children. Cochrane Database Syst. Rev..

[4] Rosenfeld R.M., Tunkel D.E., Schwartz S.R., Anne S., Bishop C.E., Chelius D.C., Hackell J., Hunter L.L., Keppel K.L., Kim A.H. (2022). Clinical Practice Guideline: Tympanostomy Tubes in Children (Update). Otolaryngol. Head. Neck Surg..

[5] Yoo M.H., Cho Y.S., Choi J., Choung Y.H., Chung J.H., Chung J.W., Han G.C., Jun B.C., Kim D.K., Kim K.S. (2022). Factors Affecting the Extrusion Rate and Complications After Ventilation Tube Insertion: A Multicenter Registry Study on the Effectiveness of Ventilation Tube Insertion in Pediatric Patients with Chronic Otitis Media with Effusion-Part II. Clin. Exp. Otorhinolaryngol..

[6] Takai S., Nomura K., Oda K., Ozawa D., Irimada M., Ikeda R., Kakuta R., Katori Y., Ohyama K. (2023). Clinical Factors Associated with the Outcomes of Long-Term Middle Ear Ventilation Tube Insertion in Pediatric Patients. Ear Nose Throat J..

[7] Mai J.P., Dumont M., Rossi C., Cleary K., Wiedermann J., Reilly B.K. (2017). Biocompatibility of “On-command” dissolvable tympanostomy tube in the rat model. Laryngoscope.

[8] Lin Y.C., Kao Y.L., Chen Y.C., Chen L.C., Dang L.H., Hung S.H. (2023). Factors Related to Ventilation Tube Extrusion Time in Children and Adults. Ear Nose Throat J..

[9] Song C.M., Park M.H., Kim Y.H., Lee J.H. (2010). Factors affecting the extrusion rate of ventilation tubes. Clin. Exp. Otorhinolaryngol..

[10] Conrad D.E., Levi J.R., Theroux Z.A., Inverso Y., Shah U.K. (2014). Risk factors associated with postoperative tympanostomy tube obstruction. JAMA Otolaryngol. Head. Neck Surg..

[11] Mickey R.M., Greenland S. (1989). The impact of confounder selection criteria on effect estimation. Am. J. Epidemiol..

[12] Otsuka S., Imai R., Kamakura T., Nishimura H., Osaki Y., Furukawa M., Yasui T., Yamashita M., Nakamura M., Iwamoto Y. (2022). How long do tympanostomy ventilation tubes last in pediatric patients with otitis media with effusion or adhesion? A study using Kaplan-Meier survival analysis. Int. J. Pediatr. Otorhinolaryngol..

[13] Almutairi A.A., Aljabr I.K., Alsindi Z.S., Alkhawajah A.A., Aljasem J.M., Alzahrani M.M., Almaqhawi A. (2025). Blood Inflammatory Markers as Predictors of Effusion Characteristics and Postoperative Hearing Outcomes in Children with Otitis Media with Effusion: A Retrospective Study. Medicina.

[14] Alvi S.A., Jones J.W., Porter P., Perryman M., Nelson K., Francis C.L., Larsen C.G. (2018). Steroid Versus Antibiotic Drops in the Prevention of Postoperative Myringotomy Tube Complications. Ann. Otol. Rhinol. Laryngol..

[15] Kay D.J., Nelson M., Rosenfeld R.M. (2001). Meta-analysis of tympanostomy tube sequelae. Otolaryngol. Head Neck Surg..

[16] Bassim M.K., Drake A.F. (2005). Tympanostomy tube obstruction related to ototopical drug therapy. Ear Nose Throat J..

[17] Alaraifi A.K., Alkhaldi A.S., Ababtain I.S., Alsaab F.A. (2022). Predictors of tympanostomy tube extrusion time in otitis media with effusion. Saudi Med. J..

[18] Szekely D., Zara F., Patrascu R., Dumitru C.S., Barb A.C., Novacescu D., Manole A., Iovanescu D., Iovanescu G. (2026). Inflammatory Endotypes of Chronic Adenoiditis and Their Impact on Persistent Middle Ear Dysfunction: A 2-Year Retrospective Translational Study Integrating Clustering and Machine Learning Approaches. Medicina.

